# Current Standards, Multidisciplinary Approaches, and Future Directions in the Management of Extrahepatic Cholangiocarcinoma

**DOI:** 10.1007/s11864-023-01153-5

**Published:** 2024-01-05

**Authors:** Margaret Wheless, Rajiv Agarwal, Laura Goff, Natalie Lockney, Chandrasekhar Padmanabhan, Thatcher Heumann

**Affiliations:** 1 Department of Medicine, Division of Hematology Oncology, Vanderbilt University Medical Center, 2220 Pierce Avenue, Preston Research Building Suite 798, Nashville, TN 37232 USA; 2 Vanderbilt-Ingram Cancer Center, Nashville, TN USA; 3 Department of Radiation Oncology, Vanderbilt University Medical Center, Nashville, TN USA; 4 Department of Surgery, Division of Surgical Oncology & Endocrine Surgery, Vanderbilt University Medical Center, Nashville, TN USA

**Keywords:** Extrahepatic cholangiocarcinoma, Biliary tract cancer, Targeted therapy, Chemotherapy, Immunotherapy

## Abstract

Biliary tract cancers are molecularly and anatomically diverse cancers which include intrahepatic cholangiocarcinoma, extrahepatic (perihilar and distal) cholangiocarcinoma, and gallbladder cancer. While recognized as distinct entities, the rarer incidence of these cancers combined with diagnostic challenges in classifying anatomic origin has resulted in clinical trials and guideline recommended strategies being generalized patients with all types of biliary tract cancer. In this review, we delve into the unique aspects, subtype-specific clinical trial outcomes, and multidisciplinary management of patients with extrahepatic cholangiocarcinoma. When resectable, definitive surgery followed by adjuvant chemotherapy (sometimes with selective radiation/chemoradiation) is current standard of care. Due to high recurrence rates, there is growing interest in the use of upfront/neoadjuvant therapy to improve surgical outcomes and to downstage patients who may not initially be resectable. Select patients with perihilar cholangiocarcinoma are being successfully treated with novel approaches such as liver transplant. In the advanced disease setting, combination gemcitabine and cisplatin remains the standard base for systemic therapy and was recently improved upon with the addition of immune checkpoint blockade to the chemotherapy doublet in the recently reported TOPAZ-1 and KEYNOTE-966 trials. Second-line all-comer treatments for these patients remain limited in both options and efficacy, so clinical trial participation should be strongly considered. With increased use of molecular testing, detection of actionable mutations and opportunities to receive indicated targeted therapies are on the rise and are the most significant driver of improved survival for patients with advanced stage disease. Though these targeted therapies are currently reserved for the second or later line, future trials are looking at moving these to earlier treatment settings and use in combination with chemotherapy and immunotherapy. In addition to cross-disciplinary management with surgical, medical, and radiation oncology, patient-centered care should also include collaboration with advanced endoscopists, palliative care specialists, and nutritionists to improve global patient outcomes.

## Introduction

Biliary tract cancers (BTC) are a diverse group of pathologically distinct entities that can be further divided into intrahepatic cholangiocarcinoma (iCCA), extrahepatic cholangiocarcinoma (eCCA), and gallbladder cancer (GBC), with the latter two of these comprising extrahepatic BTC (eBTC). Extrahepatic CCA is further divided into distal (dCCA) and perihilar cholangiocarcinoma (pCCA). Distal CCA includes the common bile duct and distal ducts whereas pCCA arises from the second-order bile ducts down to the cystic duct [1]. Given their heterogeneity, BTCs have unique pathophysiology and molecular fingerprints, which have both prognostic and treatment implications.

The mainstay of treatment for advanced disease, until the recent addition of adjunct immunotherapy, has been the chemotherapy doublet of gemcitabine (Gem) and cisplatin (Cis) [2]. With the improved detection and knowledge of actionable mutations, targeted therapy has significantly boosted the treatment armamentarium for BTCs. Herein, we review pathogenesis, molecular characterization, and treatment modalities for patients with eCCA in both early stage and advanced disease. We highlight the continued need for upfront molecular testing and clinical trial consideration across disease stages to determine the optimal timing of targeted therapy and immunotherapy and its role in combination with established therapies to improve outcomes.

## Epidemiology and prognosis

Extrahepatic CCA makes up 20–30% of all BTC in the USA and globally and is associated with certain conditions that significantly increase risk of eCCA including non-alcoholic fatty liver disease (odds ratio [OR] 2.9), cirrhosis (OR 3.8), alcohol-related liver disease (OR 2.6), and primary sclerosing cholangitis (PSC; OR 40.8), with the latter carrying up to a 36% lifetime risk of developing CCA [3–5]. Hepatitis B (HBV) and C (HCV) are associated with iCCA (predominantly) and eCCA (HBV OR 2.38; HCV OR 3.18) [4–7]. Conditions predisposing to eCCA include chronic pancreatitis, cholangitis, and choledocholithiasis [5, 8] with the estimated incidence of around 1.02 cases per 100,000 annually [9]. The prognosis for eCCA remains poor with 5-year survival estimated at 11% (localized and regional: 18%; distant: 2%) and surgery remains the only curative option in non-metastatic eCCA [10, 11]. Patients with risk factors such as higher T-stage (*p* < 0.001, locoregional disease [LRD]; *p* = 0.005, distant disease [DD] in T1a vs T1b-T4 disease) and the presence of lymphovascular invasion (*p* = 0.004, LRD; *p* = 0.01, DD) or perineural invasion (*p* = 0.04, LRD; *p* = 0.006, DD) are at risk for local and distant recurrence following definitive resection [12].

## Molecular pathogenesis and distinctive molecular characteristics of subtypes

Though classified together as BTCs, iCCA, eCCA, and GBC contain discrete molecular profiles. The differentiation between eCCA and GBC from iCCA can be traced to their cell of origin. Extrahepatic cholangiocytes derive from endoderm and are closely related to the pancreas and duodenum, whereas intrahepatic cholangiocytes arise from hepatoblasts, closely resembling hepatocytes [13]. Distal CCA can often be difficult to distinguish from ampullary and pancreatic ductal adenocarcinoma (PDAC) given their similar embryological development and overlapping mutational profile [14].

Extrahepatic CCA can be characterized into four distinct classes based on the most prevalent mutations: mesenchymal, metabolic, proliferation, and immune [15]. The mesenchymal class (47.3%) is characterized by abnormal transforming growth factor beta (TFG-β) activity and epithelial-to-mesenchymal transition leading to fibrosis and poor overall survival (OS) compared to the 3 other classes (OS HR 1.95; *p* = 0.018). Infiltration of cancer-associated fibroblasts occurs by aberrant activation of TFG-β which both suppresses immune cells in the tumor microenvironment and also promotes angiogenesis [16]. Increased cell signaling pathways such as MAPK/ERK and AKT/mTOR along with ERBB2 overexpression are increased in the proliferation class (22.5%). The metabolic class (18.7%) contains mutations associated with dysregulated bile acid metabolism (such as HDAC6 overexpression leading to ciliary loss), promoting proliferation and ultimately, metastasis [17]. Lastly, the immune class (11.5%) is characterized by infiltration of dysfunctional T-cells along with elevated PD-1 and PD-L1 expression which may represent a distinct cohort that could benefit from immunotherapy.

**Fig. 1:**
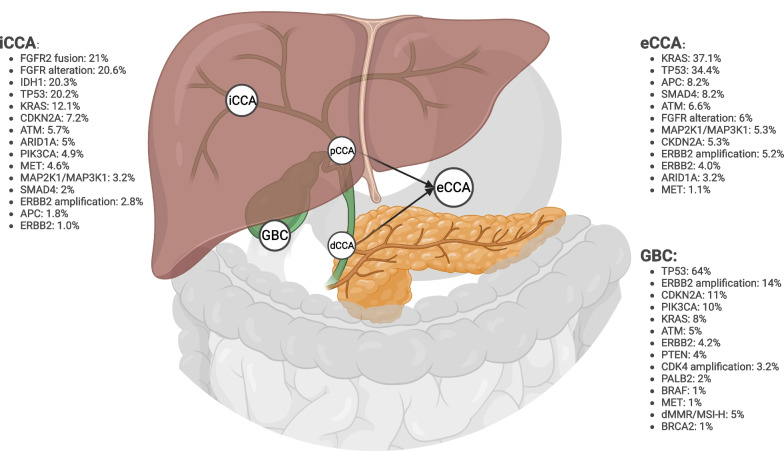
Biliary tract cancer anatomy and molecular alteration prevalence by subtype. Frequencies shown were extracted from recent BTC molecular profiling publications [23, 25•, 30, 31]. Created with Biorender.

Similar to differences at the anatomic and cellular level, molecular profiles also differ across BTC subtypes (Fig. 1). Alterations in KRAS (36.7% vs 12.1%), TP53 (34.4% vs 20.2%), APC (8.2% vs 1.6), SMAD4 (10.4% vs 2%), and ERBB2 (9.7% vs. 4.2%) are significantly more common in eCCA than iCCA [18–23]. KRAS G12D variant is most common in dCCA and GBC, followed by G12V in pCCA. Though not quite approaching the >90% incidence of KRAS mutations seen in PDAC, dCCA has the most similar mutation profile to PDAC [14, 24]. Most notably, two of the most common actionable genomic alterations with FDA-approved targeted therapies, IDH1 and FGFR2 alterations, occurring approximately 18% and 15% of iCCA, respectively, are rarely found in eCCA [15, 25•, 26, 27]. Given that up to 50% of patients with BTC have an potentially actionable mutations [28, 29] with targeted agents, guideline-recommended therapy should always include upfront molecular testing on patients with unresectable BTC [2].

## Presentation, diagnosis, and multidisciplinary review

Most patients with eCCA present with de novo metastatic disease (up to 37%) [7], as initial non-specific symptoms of fatigue, abdominal pain, and weight loss may be overlooked until disease progresses and more pressing symptoms (e.g., jaundice, acholia, biliary obstruction) develop [9]. Biliary obstruction leads to imaging and subsequent diagnosis earlier in eCCA than iCCA due to external biliary compression [4]. Computed tomography (CT) is the imaging modality of choice except in pCCA where magnetic resonance cholangiopancreatogram (MRCP) can be especially useful to delineate biliary tree anatomy and determine local invasion [9]. Prior to obtaining pathology, PDAC may be difficult to distinguish from dCCA based on imaging alone [32].

After imaging is obtained, endoscopic retrograde cholangiopancreatography (ERCP) is often used to directly visualize the biliary tree, obtain brush cytology, biopsy, and relieve obstruction with biliary stenting, if needed [9]. Initial diagnosis of CCA based on ERCP (including brush cytology and biopsy via forceps) may be difficult as the sensitivity and specificity from ERCP are reported as 49% and 96%, respectively. EUS with FNA of biliary stricture has slightly improved sensitivity and specificity of 75% and 100%, respectively, but cannot exclude CCA due to low sensitivity [33]. Fluorescence in situ hybridization (FISH), an alternative method examining chromosomal and 9p21 abnormalities, can be combined with cytology to improve sensitivity (35 to 63% [with FISH]; *p* < 0.05) [34].

Patients should be presented in a multidisciplinary tumor board since treatment modalities range from surgery, locoregional treatment, systemic therapy, and can often involve a combinatorial approach. Implementation of multidisciplinary tumor boards has been shown to prolong life, improve patient-reported quality of life outcomes, and increase clinical trial awareness and evaluation [35]. Multidisciplinary patient care also should extend to involving dieticians for nutrition needs, palliative care for symptom management and serious illness conversations, and interventionalists for endobiliary decompression, as indicated.

## Early-stage disease

### Surgical considerations and recurrence risk factors

For patients who present with localized disease, definitive treatment with surgical resection can offer cure. Unfortunately, only 20% of patients have resectable disease at presentation, and recurrence rates up to 75% have been reported [6, 36].

If an anatomical and biological candidate, surgery is often extensive and usually requires a pancreaticoduodenectomy (Whipple procedure) for dCCA and extensive hepatectomy for pCCA to achieve R0 resection [37]. Underlying liver disease or bilateral eCCA can be contraindications to surgery. Liver function may need to be optimized by relieving biliary obstruction prior to patients undergoing resection. In those with underlying liver disease or bilateral biliary duct involvement by eCCA without lymph node (LN) or metastatic disease, liver transplant can be considered and will be discussed subsequently with neoadjuvant therapy [38]. Higher rates of recurrence after surgical resection are seen in patients with vascular invasion, higher tumor stage (5-year OS 28% [T2b/T3] vs 57% [T1–2a]; HR 2.23; 95% CI 1.24–4.01; *p* = 0.007), nodal spread (5-year OS 27% [Node +] 50% vs 27% [Node −], HR 2.07 [95% CI 1.16, 3.68], *p* = 0.014), R1 resection (5-year OS 13% [R1] vs 49% [R0], HR 2.09 [95% CI 1.17, 3.71], *p* = 0.012), and initial CA19-9 greater than 37 U/mL (HR 1.42) [39–42].

Carbohydrate antigen 19-9 (CA19-9) can offer predictive value as a surrogate marker for micrometastatic disease and post-operative recurrence risk. Elevated CA19-9 ( ≥37 U/mL) are found in 59.1% of CCA [43] with a reported sensitivity and specificity of 66% and 88%, respectively [44]. A retrospective review analyzed CA19-9 in patients with eCCA (*N* = 390) before and after curative-intent surgery who had either normal (<37 U/mL) or elevated (≥ 37 U/mL) values. In patients with normal values prior to surgery or elevated values that normalized postoperatively, the 5-year OS was significantly better than patients who had elevated CA19-9 both pre- and post-operatively (53%, 38%, 23%, respectively; *p* <0.001). Patients with CA19-9 <37 U/mL prior to surgery had a significantly better OS with R0 resection (5-year OS 59% vs 7%; *p* < 0.001). Even though the rates of local recurrence were similar, patients with normal CA19-9 serum level were less likely to have distant recurrence than patients with pre-operatively elevated (*p* = 0.003) or persistently elevated post-operative values (*p* < 0.001). CA19-9 should always be obtained prior to surgery given its use in evaluating micrometastatic disease and risk of recurrence after resection [42].

### Adjuvant therapy

#### Adjuvant chemotherapy

Prior to 2019, adjuvant chemotherapy had not demonstrated a benefit over observation in resected BTC. The phase III PRODIGE12/ACCORD18 trial [41] comparing adjuvant GemOx to observation in resected BTC patients showed no significant differences in RFS and OS. However, with the reporting of the BILCAP trial [45], 6 months of adjuvant Cap became standard of care. This phase III study randomized 447 patients (*n* = 284 [64%] eCCA; *n* = 279 [62%] R0 resection) with resected BTC to either adjuvant Cap (D1–14, 21-day cycles) or observation for 6 months. On per protocol analysis, adjusting for disease grade and nodal status, Cap significantly improved OS (mOS 49.6 months [Cap] vs 36.1 months [obs]; HR 0.74, [95% CI 0.59, 0.94]) and DFS (mDFS 25.3 months [Cap] vs 16.8 months [obs]; DFS HR 0.77 [95% CI 0.61, 0.97]) compared to observation. Recently, the phase II STAMP trial [46] randomized patients with resected lymph node-positive (LN-positive) eCCA to either GemCis or Cap (*n*=101), but failed to demonstrate the benefit of GemCis over Cap in OS (HR 1.08 [95% CI 0.71, 1.64], *p* = 0.404) or DFS (HR 0.96 [95% CI 0.71, 1.3], *p* = 0.430) with GemCis having an 84% grade 3+ AE rate (vs 14% with Cap). The currently ongoing phase III ACTICCA-1 trial (NCT02170090) [47] will compare adjuvant GemCis with observation, the results of which are expected in 2024 (see Table 1 for a summary of seminal adjuvant therapy clinical trials for BTC).

**Table 1:** Summary of select biliary tract cancer clinical trials conducted in the adjuvant, first-line advanced stage, or second-line advanced stage treatment setting

NCT	Trial	Study agent	Study population	Primary outcome	mPFS, RFS, or DFS (months)	HR (95% CI) *p*-value	mOS (months)	HR (95% CI) *p*-value	ORR (%)
Adjuvant therapy									
NCT00363584	BILCAP [48]	Cap vs obs	Resected BTC	OS	DFS: 25.9 vs 17.4	0.75 (0.58, 0.98) *p* = 0.033	49.6 vs 36.1	0.71 (0.55, 0.92) *p* = 0.010	
							GBC: 0.84 (0.43, 1.63) dCCA: 0.70 (0.47, 1.06) pCCA: 1.08 (0.68, 1.71)		
NCT03712605	STAMP [46]	GemCis vs Cap	Resected, LN-positive eCCA	DFS	DFS: 14.3 vs 11.1	0.96 (0.71–1.30) p = 0.86	35.7 vs 35.7	1.08 (0.71, 1.64) *p* = 0.404	
					pCCA: 1.38 (CI 0.91, 2.11) dCCA: 0.63 (0.42, 0.96)		pCCA: 1.50 (0.84, 2.65) dCCA: 0.75 (CI 0.41, 1.38)		
NCT01313377	PRODIGE 12-ACCORD 18-UNICANCER GI [41]	GemOx vs obs	Resected BTC	RFS	RFS: 30.4 vs 18.5	0.88 (0.62, 1.25) *p* = 0.48	75.8 vs 50.8	1.08 (CI 0.79, 1.66) *p* = 0.74	
					GBC: 2.56 (CI 1.04, 6.23) eCCA: 0.60 (CI 0.34, 1.1)		GBC: 3.4 (1.17, 9.8) eCCA: 0.70 (0.34, 1.44)		
NCT02170090	ACTICCA-1 [47]	GemCis vs observation	Resected BTC	DFS	Ongoing				
NCT00789958	SWOG S0809 [49••]	GemCap followed by CRT with Cap	Resected eCCA or GBC with pT2-4, N1, or R1 disease	2-yr OS	2-yr DFS: 52% R0: 54% R1: 48% GBC: 48% eCCA: 54%		2-yr OS: 65% R0: 67% R1: 60% GBC: 56% eCCA: 68%		
Advanced first line (chemotherapy)									
NCT00262769	ABC-02 [50]	GemCis vs Gem	1st line, advanced BTC	OS	mPFS: 8.0 vs 5.0	0.63 (0.51, 0.77) *p* < 0.001	11.7 vs 8.1	0.64 (0.51, 0.8) *p* < 0.001	81.4% vs 71.8% *p* = 0.049
NCT03768414	SWOG-1815 [51]	GAP vs GemCis	1st line, advanced BTC	OS	mPFS: 8.2 vs 6.4	0.92 (85% CI 0.72, 1.16) *p* = 0.47	14 vs 12.7	0.93 (0.74, 1.19) *p* = 0.58	34% vs 25% *p* = 0.11
		GemOx[52]	1st line, advanced BTC	ORR	mPFS: 3.4 GBC: 2.5 CCA: 3.8	(2.5, 4.6) (1.6, 4.3) (2.7, 5.6)	8.8 GBC: 6.1 CCA: 11	(6.9, 11.1) (3.0, 10.8) (7.5, 14.1)	GBC: 4.3% CCA: 20.5%
		GemCap[53]	1st line, advanced BTC	ORR, DOR, OS	mPFS: 7.0 GBC: 4.4 CCA: 9.0	(4.6, 11.8)	14.0	(7.3 to NE)	31% GBC: 27% CCA: 34%
NCT02591030	PRODIGE 38 AMEBICA [54]	mFOLFIRINOX vs GemCis	1st line, advanced BTC	6-mo PFS	mPFS: 6.2 vs 7.4		11.7 vs 13.8		25% vs 19.4%
CRTI/2010/091/001406 [55]		mGemOx vs GemCis	1st line, advanced GBC	OS	mPFS: 5.0 vs 4.0	*p* = 0.047	9 vs 8.3	0.057 (0.6, 1.01) *p* = 0.057	25.2% vs 23.4%
Advanced first line (chemoimmunotherapy)									
NCT03875235	TOPAZ-1 [56••]	GemCis+durva vs Gemcis+placebo	1st line, advanced BTC	OS	mPFS: 7.2 vs 5.7	0.75 (0.63, 0.89) *p* = 0.001	12.8 vs 11.5	0.80 (0.66, 0.97) *p* = 0.021	26.7% vs 18.7%
NCT04003636	KEYNOTE-966 [57••]	GemCis+pembro vs GemCis	1st line, advanced BTC	OS	mPFS: 6.5 vs 5.6	0.86 (0.75, 1.0) *p* = 0.023	12.7 vs 10.9	0.83 (0.72, 0.95) *p* = 0.0034	29% vs 29%
NCT 04677504	IMbrave-151 [58]	GemCis+atezo + bev vs GemCis+atezo	1st line, advanced BTC	PFS	mPFS: 8.4 vs 7.9	0.76 (0.51, 1.14)	Ongoing		24% vs 25%
Advanced second line									
NCT01926236	ABC-06 [59••]	FOLFOX vs ACS	2nd line, advanced BTC	OS	mPFS: 4.0 (FOLFOX)		6.2 vs 5.3	0.69 (0.5, 0.97) p = 0.031	5% (FOLFOX)
NCT03524508	NIFTY[60]	FOLFIRI vs 5FU	2nd line, advanced BTC	PFS	mPFS: 7.1 vs 1.4 GBC: 9 vs 1.4 eCCA: 8.8 vs 1.6	HR 0.56 (0.39, 0.81) *p* = 0.0019 GBC: 0.3 (0.15, 0.61) eCCA: 0.79 (0.38, 1.66)	8.6 vs 5.5 GBC: 12.4 vs 4.7 eCCA: 8.8 vs 7.6	0.68 (CI 0.48, 0.98) *p* = 0.035 GBC: 0.29 (0.14, 0.58) eCCA: 1.06 (0.53, 2.16)	14.8% vs 5.8%
NCT03043547	NALIRICC [61]	FOLFIRI vs 5FU	2nd line, advanced BTC	PFS	mPFS: 2.76 vs 2.3		6.9 vs 8.21		14.3% vs 3.9%

Abbreviations: *BTC*, biliary tract cancer; *CCA*, cholangiocarcinoma; *DOR*, duration of response; *eCCA*, extrahepatic cholangiocarcinoma; *GBC*, gallbladder cancer; *ORR*, objective response rate; *mOS*, median overall survival; *mPFS*, median progression-free survival; *NE*, not evaluable

#### Adjuvant chemoradiation

In a meta-analysis of 20 studies comparing outcomes between BTC patients who received adjuvant chemotherapy (CT), chemoradiotherapy (CRT), or radiotherapy (RT) after resection, overall, patients who received CT (OR 0.39) or CRT (OR 0.61) had improved OS compared to those who received RT alone (OR 0.98) [62]. When analyzed by margin-positivity and LN-positive status, most patients with R1 resections received RT alone (63% of patients; OR 0.33 [95% CI 0.14, 0.81], *p* = 0.01) which was associated with significantly improved OS, whereas no benefit was seen with RT in R0 resections (OR 1.26 [95% CI 0.88, 1.79], *p* = 0.20) [62]. In LN-positive disease, 77% of patients received adjuvant CT while the remaining received CRT in which showed an overall OS benefit with adjuvant therapy (OR 0.49 [95% CI 0.3, 0.8], *p* = 0.002) [62]. A more recent meta-analysis evaluated 5-year OS in patients who received adjuvant RT compared to observation and found significantly improved OS (OR 0.63 [95% CI 0.5, 0.81], *p* = 0.0002) and lower rate of local recurrence (OR 0.54 [95% CI 0.38, 0.76], *p* = 0.0004) in LN-positive disease (OR 0.15 [95% CI 0.07, 0.35], *p* < 0.00001) and R1 resection (OR 0.4 [95% CI 0.19, 0.85], *p* = 0.0002) with RT [63]. Additionally, a National Cancer Database analysis including patients with R1 resection or LN-positive disease who received adjuvant CT had significantly improved OS with the addition of adjuvant RT (mOS 34 vs 27 months; *p* < 0.001) [64].

The single-arm phase II trial, SWOG S0809 [49••], explored the role of four adjuvant cycles of GemCap followed by concurrent CRT (systemic Cap with 45 Gy to regional lymphatics; 54 to 59.4 Gy to tumor bed) in resected eCCA and GBC (*N* = 78; *n* = 54 [eCCA]). This demonstrated a mOS of 35 months with 2-year RFS of 54% and 11% local relapse rate. The 2-year OS of 65% (67% [R0]; 60% [R1]) was significantly higher than the rates expected based on historical controls. The mOS of 35 months was similar between patients with R0 and R1 resections which suggests that this approach could bring the risk of recurrence with R1 resection closer to that associated with R0 resections. Although not powered for comparison, the similar OS may suggest efficacy of the treatment regimen, particularly in patients with R1 resections [36]. Reported grade 3/4 AEs included neutropenia (35%), hand-foot syndrome (13%), diarrhea (8%), and lymphopenia (8%) [49••]. Based on these findings, the NCCN recommends adjuvant CT in R0 resection and either CT and/or CRT in R1 or LN-positive disease under the guidance of a multidisciplinary team [2].

### Locally advanced disease

#### Upfront/perioperative systemic therapy

For patients with locally advanced/potentially resectable disease, the use of upfront therapy prior to surgery to increase curative resection rates has been increasing over time, but its role remains unclear [65, 66]. The benefits of upfront/neoadjuvant therapy include downstaging tumor burden, systemic therapy treatment prior to major surgery and potential post-operative recovery complications/functional setbacks, and testing disease biology (in terms of pathological assessment of treatment response and conversely avoiding an unnecessary surgery for patients whose cancer progresses while upfront therapy) [67]. A retrospective review of resectable dCCA patients who underwent either neoadjuvant therapy followed by resection or upfront resection showed more R0 resection rates (83% vs 76%; *p* = 0.04) and improved mOS (38.4 vs 25.6 months; *p* < 0.001) in patients who received neoadjuvant therapy compared to upfront surgery [68]. Another review including 45 patients with eCCA (*n* = 33 [adjuvant RT]; *n* = 12 [neoadjuvant CT]) found that 11 (91%) patients treated neoadjuvantly had R0 resections and numerically improved 5-year OS, trending towards significance (53% vs 23%; *p* = 0.16) compared to those who received adjuvant treatment [69].

The ongoing phase III GAIN trial [70] in Germany is comparing the use of perioperative GemCis (3 cycles before and after surgery) in patients with incidentally discovered GBC and resectable or borderline resectable eCCA/iCCA to upfront surgery followed by adjuvant therapy (physician’s choice). The primary outcome of OS is anticipated in 2024 as this will be the first phase III trial comparing perioperative therapy to upfront surgery [71]. In patients with locally advanced or borderline resectable disease, the use of upfront therapy for downstaging, improving R0 resection rates, and allowing response to therapy should be considered, but prospective data is needed [69].

#### Chemoradiotherapy and liver transplantation

Upfront CRT followed by liver transplantation may be considered in a select pCCA cases without nodal or metastatic disease and who are not candidates for standard resection [72]. In a series of 11 pCCA patients who subsequently underwent liver transplant as their definitive therapy, mOS was 25 months (range 4–174) [73]. A larger study then included 56 pCCA patients who received neoadjuvant RT, brachytherapy, and fluorouracil (5FU) prior to transplant. Of the 28 patients who underwent transplantation, 1-year OS was 88% and 5-year OS was 82% [74]. A recent review found that patients with LN-negative, unresectable pCCA who received upfront CRT followed by transplant had significantly better 5-year DFS when compared to matched LN-negative, resectable patients who underwent upfront resection (50.2% vs 17.4%; *p* < 0.001) [75••]. Several high-volume transplant centers have implemented institutional protocols [76] to identify and treat select pCCA patients with this high-impact intervention.

## Advanced stage disease

In addition to the discussion below, the highlighted clinical trials in advanced-stage BTC (all-comers population) are summarized in Table 1.

### First line — chemotherapy

#### Gemcitabine and cisplatin

Until recently, standard-of-care (SOC) first-line therapy for unresectable eCCA was based on the results of the seminal phase III ABC-02 trial which randomized 410 patients with locally advanced or metastatic BTC to Gem (*N* = 204) or GemCis (*N* = 206) [50]. Compared to patients who received Gem alone, treatment with the GemCis doublet significant improved both OS (mOS 11.7 months [GemCis] vs. 8.1 months [Gem]; HR 0.64 [95% CI 0.51, 0.8], *p* < 0.001) and PFS (mPFS 8.0 months [GemCis] vs. 5.0 months [Gem]; HR 0.63 [95% CI 0.51, 0.77], *p* < 0.001). Observed ORR favored GemCis (26.1% vs. 15.5%). The clinical benefits of the chemotherapy doublet were observed across all BTC subtypes including eCCA (eCCA OS HR = 0.73 [95% CI 0.43, 1.23]) and were similar to the overall population. Despite a numerically higher incidence of grade 3+ neutropenia in the doublet group (25.3% [GemCis] vs. 16.6% [Gem]), incidences of grade 3+ infection were similar between the two arms (18.2% [GemCis] vs. 19.1% [Gem]). Based on these results, GemCis became SOC first-line (1L) therapy for BTC in 2010 [2].

#### Gemcitabine, cisplatin, and albumin-bound paclitaxel

After promising results in a single-arm phase II trial [77•], SWOG 1815 [51] examined whether 1L GemCis could be improved with the addition of a third chemotherapeutic agent, albumin-bound (nab) paclitaxel (GAP). Study participants were randomized 2:1 to GAP (*n*=294) or standard GemCis (*n*=147). Treatment with GAP did not provide a statistically significant benefit in OS (mOS 14.0 [GAP] vs. 12.7 months [GemCis]; HR 0.93 [95% CI 0.74, 1.19], *p* = 0.58), PFS (mPFS 8.2 months [GAP] vs. 6.4 months [GemCis], HR 0.92 [95% CI 0.72, 1.16], *p* = 0.47), or ORR (31% [GAP] vs. 22% [GemCis]) compared to the standard doublet. Notably, GAP treatment had significantly more grade 3+ hematologic toxicity compared to the GemCis (60% vs. 45%, *p* = 0.003) and had numerically higher discontinuation rates due to toxicity (24% [GAP] vs. 19% [GemCis]). For now, GemCis continues to be the chemotherapy backbone of choice for eCCA.

#### Gemcitabine and oxaliplatin

For patients with chronic kidney disease where treatment with cisplatin would be contraindicated due to the potential for cisplatin-related nephrotoxicity, GemOx is a suitable alternative. GemOx (dosed as Gem 1000mg/m^2^ + Ox 100mg/m^2^ [D1, q14 days]) was studied in a single-arm phase II trial [52] in 67 patients with unresectable BTC (*n* = 13 [eCCA]) with a mOS of 11.0 months for non-GBC BTC. GemOx was overall well-tolerated and has been associated with less neutropenia and thrombocytopenia than GemCis although grade 3/4 AEs of thrombocytopenia (14.9%) and neutropenia (12%) were reported [52]. Modified (m)GemOx (Gem 900 mg/m^2^ + Ox 80 mg/m^2^ × 6 cycles) has been found to significantly improve clinical outcomes in unresectable GBC patients compared to best supportive care (BSC) or 5FU (425mg/m^2^ weekly bolus): median OS of 4.5, 4.6, and 9.5 months for BSC, 5FU, and mGemOx, respectively (*p*=0.039); mPFS of 2.8, 3.5, and 8.5 months for BSC, 5FU, and mGemOx, respectively (*p* < 0.001) [78]. A subsequent phase III study in 1L unresectable GBC (*N* = 243) compared mGemOx (Gem 900 mg/m^2^ + Ox 80 mg/m^2^ [days 1 and 8, q21 days, max 6 cycles]) with GemCis (Gem 1000mg/m^2^ + Cis 25 mg/m^2^ 8 cycles [days 1 and 8, q21 days, max 8 cycles]). OS was similar between the two cohorts at 9 and 8.3 months in the mGemOx and GemCis groups, respectively (HR 0.78 [95% CI 0.60, 1.01], *p* = 0.057). However, this study was not powered to assess for superiority. Significantly less grade 3+ nephrotoxicity was reported with mGemOx (0% [mGemOx] vs. 7% [GemCis], *p* = 0.01), numerically less grade 3+ neutropenia (18% [mGemOx] vs. 26% [GemCis], *p* = 0.12) but with the tradeoff of increased grade 3+ neuropathy (8% [mGemOx] vs. 1% [GemCis], *p* = 0.02) [55].

#### Gemcitabine and capecitabine

For patients with existing neuropathy, e.g., end organ complications from diabetes mellitus, Gem in combination with capecitabine (GemCap) can be considered. In 2005, a phase II trial evaluated GemCap in 45 patients with advanced BTC (*n*=23 [CCA]) in the 1L with reported mOS of 14 months (19 months [CCA] vs. 6.6 months [GBC], *p* = 0.011), mPFS of 7 months (9.0 months [CCA] vs. 4.8 months [GBC], *p* = 0.026), ORR 31% [34% [CCA] vs. 28% [GBC]), and DOR 13.8 months. There was a significant OS (HR 3.61 [95% CI 1.35, 9.67], *p* = 0.11) and PFS (HR 2.37 [95% CI 1.11, 5.06], *p* = 0.026) benefit seen in CCA compared to GBC [53]. This combination was generally well tolerated, and grade 3/4 AEs included neutropenia (34%), thrombocytopenia (11%), and hand-foot rash (9%) [53].

#### Modified FOLFIRINOX

The 2022 phase II/III PRODIGE 38 AMEBICA trial [54] compared GemCis with modified (no bolus) 5FU, leucovorin, irinotecan, and oxaliplatin (mFOLFIRINOX) in 191 treatment-naïve advanced BTC patients. Because the phase II portion did not meet its 6-month PFS goal (44.6% [mFOLFIRINOX] vs 47.3% [GemCis]), it was not expanded to phase III. There was no significant overall difference in mPFS (6.2 months [mFOLFIRINOX] vs 7.4 months [GemCis]) or mOS (11.7 months [mFOLFIRINOX] vs 13.8 months [GemCis]). In a sub-group analysis, there was a trend towards better 6-month PFS with mFOLFIRINOX in patients with eCCA (HR 1.29 [95% CI 0.65, 2.57], *p* = 0.47) compared to iCCA patients suggesting that mFOLFIRINOX may have more benefit in eCCA. Taking into consideration the similar molecular profiles of dCCA and PDAC, mFOLFIRINOX could be considered in fit patients with eCCA; however, further prospective data in this select patient population are needed.

### First line — chemoimmunotherapy

#### Gemcitabine, cisplatin, and durvalumab

Despite underwhelming clinical outcomes with immunotherapy-exclusive regimens in BTC [79–82], immune checkpoint inhibitor (ICI) therapy, when added to first-line chemotherapy, has recently led to the first change in SOC systemic therapy in BTC in more than a decade. The phase III TOPAZ-1 trial [56••] randomized 685 patients (*n* = 383 [iCCA], *n* = 131 [eCCA], *n* = 171 [GBC]) to GemCis with or without the programmed cell death ligand 1 (PD-L1) inhibitor, durvalumab (Durva). Patients received GemCis ± Durva (21-day cycles, max 8 cycles) and then were continued on either Durva monotherapy or placebo monthly until progression or intolerance. Compared to standard GemCis, patients treated with GemCis + Durva had significantly improved OS (mOS 12.8 months [GemCis + Durva] vs. 11.5 months [GemCis], HR 0.80 [95% CI 0.66, 0.97], *p* = 0.21), PFS (mPFS 7.2 months [GemCis + Durva] vs. 5.7 months [GemCis], HR 0.75 [95% CI 0.63, 0.89], *p* = 0.001), and ORR (26.7% [GemCis + Durva] vs. 18.7% [GemCis]) with a small amount of grade 3+ immune-related toxicity (2.4%). When broken down by tumor site, OS benefit of the GemCis + Durva in eCCA patients (OS HR 0.61 [95% CI 0.41, 0.91]) was similar to iCCA and more pronounced than GBC patients. Benefit was also independent of PD-L1 status with CPS <1% and ≥1% having similar outcomes. Based on these significant results, GemCis + Durva was approved by the FDA as a 1L option in 2022 for advanced or metastatic BTC.

#### Gemcitabine, cisplatin, and pembrolizumab

Shortly following the TOPAZ-1 trial and its 1L FDA-approval, the phase III KEYNOTE-966 trial [57••], assessing GemCis with or without programmed cell death protein 1 (PD-1) inhibitor, pembrolizumab (Pembro), was reported (*n* =1069 [19% eCCA]). In contrast to TOPAZ-1, KEYNOTE-966 allowed for the continuation of Gem as part of the maintenance regimen alongside Pembro or placebo, which was a critique of TOPAZ-1 which only compared Durva vs placebo in the maintenance setting. Additionally, KEYNOTE-966 had better representation of sites outside of East Asia (55%). KEYNOTE-966 met its primary endpoint with a mOS of 12.7 months with GemCis + Pembro vs 10.9 months in the SOC arm (HR 0.83 [95% CI 0.72, 0.95], *p* = 0.0034). This benefit was similarly independent of PD-L1 status (CPS of <1% or ≥1%). In the prespecified subgroup analysis for OS, GemCis and GemCis + Pembro had the most benefit in iCCA compared to eCCA (HR 0.99 [95% CI 0.73, 1.35]). Unlike TOPAZ-1, KEYNOTE-966 did not demonstrate a significant additive benefit of Pembro when it came to PFS (mPFS 6.5 months [GemCis + Pembro] vs. 5.6 months [GemCis]; HR 0.86 [0.75, 1], *p* = 0.23) or ORR (29%, both arms) [57••]. GemCis + Pembro is under accelerated FDA review for a 1L indication. Taken together, TOPAZ-1 and KEYNOTE-966 have solidified the role of immune checkpoint blockade as an adjunct to standard chemotherapy in the 1L management strategy of advanced BTCs. However, the benefit remains modest and more optimal predicative biomarkers are needed to identify patients and tumors that will benefit most from the addition of ICI.

#### Gemcitabine, cisplatin, atezolizumab, and bevacizumab

The immunosuppressive effect of tumor cells can be attributed in part to aberrant vascular endothelial growth factor (VEGF) expression. Abnormal VEGF activity causes dysfunctional angiogenesis which prevents proper T-cell infiltration and creates a tumor microenvironment exempt from immune regulation [83]. The addition of VEGF inhibitors, like bevacizumab (Bev), to ICI has been postulated to further increase immune response by creating neo-antigens to enhance T-cell recognition and anti-cancer activity. Based on this, the IMbrave151 phase II trial [58] randomized 162 patients (19% eCCA) to GemCis + Atezo ± Bev (21-day cycles). GemCis + Atezo ± Bev was given for a maximum of 8 cycles and Atezo ± Bev was subsequently continued until disease progression or toxicity. Initial results showed similar rates of grade 3+ AEs, and a modest improvement in PFS of 8.4 months with GemCis + Atezo + Bev compared to 7.9 months with GemCis + Atezo. While ORR was similar between the arms (24% and 25%), 89% of patients treated with Bev had a duration of response (DOR) ≥6 months compared to 47% without Bev, suggesting there is potentially a more durable anti-cancer effect with the addition of VEGF inhibition to ICI [58]. However, further follow-up and mature OS outcomes are needed.

### Second line — chemotherapy

#### FOLFOX

The phase III ABC-06 trial [59••] prospectively compared FOLFOX (5FU [bolus + extended infusion], leucovorin, Ox) plus active symptom control (ASC) to ASC alone in advanced BTC with prior progression on 1L Gem-based chemotherapy (*N* = 162; *n* = 45 [eCCA]). The addition of FOLFOX to ASC modestly improved OS (mOS 6.2 months vs 5.3 months; HR 0.69 [95% CI 0.5, 0.97], *p* = 0.031) which was less pronounced in eCCA (HR 0.84 [95% CI 0.45–1.56]) compared to the overall population, though not statistically significant. The mPFS was 4 months and ORR was 5% with FOLFOX; however, the incidence of grade 3/4 infection (16%) was greater with FOLFOX. Six-month and 1-year survival rates were 50.6% and 25.9% for the FOLFOX arm compared to 35.5% and 11.4% for ASC [59••].

#### Liposomal irinotecan/5-FU and FOLFIRI

The South Korean phase IIb NIFTY trial [84] evaluated 5FU-leucovorin with nanoliposomal irinotecan (nal-IRI) compared to 5FU alone after progression on GemCis (*N* = 174; *n* = 47 [eCCA]). Unlike ABC-06, NIFTY was powered for PFS as the primary endpoint. Updated analysis revealed a significantly improved OS (mOS 8.6 months [nal-IRI + 5FU] vs 5.3 months [5FU], HR 0.68 [95% CI 0.48, 0.95], *p* = 0.02), PFS (mPFS 4.2 months [nal-IRI + 5FU] vs 1.7 months [5FU], HR 0.61 [95% CI 0.44, 0.86], *p* = 0.004), and ORR (12.5% [nal-IRI + 5FU] vs 3.5% [5FU]) favoring the study doublet. There were no significant differences in outcomes of eCCA patients compared to other subgroups. There was more grade 3/4 neutropenia (24% [nal-IRI + 5FU] vs 1% [5FU]) and anemia (9% [nal-IRI + 5FU] vs 3% [5FU]) in study doublet arm [84].

The German phase II NALIRICC [61] trial (*N* = 100, *n* = 19 [eCCA]) similarly compared nal-IRI + 5FU vs 5FU but did not find the same improvement in OS (mOS 8.21 months [5FU] vs 6.9 months [5FU + nal-IRI]; HR 1.08 [95% CI 0.68, 1.72]) or PFS (mPFS 2.3 months [5FU] vs 2.76 months [5FU + nal-IRI]; HR 0.87 [95% CI 0.68, 1.72]) [61]. These discordant results may be explained by different patient populations (NALIRICC in a European population, NIFTY in an Asian population) along with more iCCA patients included in NALIRICC than NIFTY (64% vs 43%) [85].

Standard irinotecan + 5FU (FOLFIRI) was compared against mFOLFOX in a phase II trial [86] in 2L advanced BTC patients (*N* = 118; *n* = 29 [eCCA]). Both arms had similar OS (mOS 6.3 months [mFOLFOX] vs 5.7 months [mFOLFIRI], HR 1.1 [95% CI 0.7, 0.16], *p* = 0.677), PFS (mPFS 2.8 months [mFOLFOX] vs 2.1 months [mFOLFIRI], HR 1.0 [95% CI 0.7, 1.5], *p* = 0.974), and ORR (5.9% [mFOLFOX] vs 4.0% [mFOLFIRI]). Patients who received mFOLFOX had more grade 3+ thrombocytopenia (10.7% vs 8.6%) and peripheral neuropathy (3.6% vs 1.7%), and thus, mFOLFIRI is a reasonable option given comparable efficacy for patients who have residual chemo-induced neuropathy or thrombocytopenia from previous therapy [86]. 5FU monotherapy is also an option with lower rates of grade 3/4 AEs as shown in the NALIRICC study (70.8% [5FU + nal-IRI] vs 50% [ 5FU]), though with limited efficacy [61]. FOLFOX, FOLFIRI, nal-IRI + 5FU, and 5FU are all recommended by the NCCN in the 2L given their comparable efficacies and unique side effect profiles [2]. Overall, second-line therapy chemotherapy options are available, but the magnitude of clinical benefit is limited (Table 1), highlighting the importance of molecular sequencing to identify potentially actionable mutations (Fig. 1, Table 2) and need for impactful clinical trials in this space.

**Table 2:** Summary of select clinical trials evaluating targeted therapy in biliary tact cancer patients harboring actionable genetic alterations

NCT	Trial	Study agent	Study population	Primary outcome	mPFS (months)	HR (95% CI) *p*-value	mOS (months)	HR (95% CI) *p*-value	ORR (%)
ERBB2/HER2									
NCT01953926	SUMMIT [87]	Neratinib	*HER2* expressing, advanced BTC	ORR	2.8	(1.1, 3.7)	5.4	(3.7, 11.7)	16%
NCT02091141	MyPathway [88]	Pertuzumab, trastuzumab	*HER2* expressing, 2nd line, advanced BTC	ORR	4.0 GBC: 4.2 eCCA: 6.8	(1.8, 5.7) (1.8, 8.2) (1.3, 13.5)	10.9 GBC: 14.2 eCCA: 8	(5.2, 15.6) (4.2, NE) (2.0, NE)	23%
JMA-IIA00423	HERB [89]	Trastuzumab-deruxtecan	*HER2* expressing, 2nd line, advanced BTC	ORR	4.4	(2.8, 8.3)	7.1	(4.7, 14.6)	36.4% *p* = 0.01
NCT04482309	DESTINY-PanTumor02 [90]	Trastuzumab-deruxtecan	*HER2* expressing, advanced, solid tumors	ORR	Ongoing				37.1% BTC: 22%
NCT04466891	HERIZON-BTC-01 [91••]	Zanidatamab	*HER2* expressing, 2nd line, advanced BTC	ORR	5.5	(3.7, 7.2)	Ongoing		41.3% GBC: 46.3 eCCA: 43.8%
NCT04579380	SGNTUC-019 [92]	Tucatinib + trastuzumab	*HER2* expressing, 2nd line, advanced BTC	ORR	5.5	90% CI 3.9, 8.1	Ongoing		46.7%
BRAF^V600E^									
NCT02034110	ROAR [93]	Dabrafenib + trametinib	BRAF^V600E^ mutated solid tumors	ORR	8.9	(5.6, 13.7)	13.5	(10.4, 17.6)	47%
KRAS^G12C^									
NCT03785249	KRYSTAL-1 [94]	Adagrasib	KRAS^G12C^ mutated solid tumors	ORR	8.6	(2.7, 11.3)	15.1	(8.6, NE)	41.7%
EGFR									
NCT00552149	BINGO [95]	GemOx + cetuximab vs GemOx	1st line, advanced BTC	4-mo PFS	6.1 vs 5.5		11 vs 12.4		18% vs 17%
NCT01149122 [96]		GemOx vs GemOx + erlotinib	1st line, advanced BTC	PFS	4.2 vs 5.8	0.80 (0.61, 1.03) *p* = 0.087	9.5 vs 9.5	0.93 (0.69, 1.25) *p* = 0.611	16% vs 30%
MSI-H/dMMR									
NCT02628067	KEYNOTE-158 [97]	Pembrolizumab	MSI-H/dMMR advanced solid tumor, 2nd line	ORR	CCA: 4.2	(2.1–NE)	CCA: 24.3	(6.5, NE)	CCA: 40.9%
TMB-H									
NCT03668119	CHECKMATE-848 [98]	Ipi-nivo	TMB-H advanced solid tumors, 2nd line stratified by blood or tumor tissue TMB-H	ORR	4.1	( 2.8, 11.3)	14.5	(7.7, NE)	35.3%
LAT1									
UMIN000034080 [99••]		Nanvuranlat	2nd line or later, advanced BTC	PFS	0.56 (0.34, 0.90) *p* = 0.016 LAT1-h: 0.44 (0.23, 0.85) *p* = 0.013 LAT1-l: 1.44 (0.56, 3.69) *p* = 0.444		0.85 (0.53, 1.36) *p* = 0.493 LAT1-h: 0.67 (0.35, 1.30) *p* = 0.231 LAT1-l: 1.5 (052, 4.34) *p* = 0.454		
FGFR2 fusion, rearrangement									
NCT02924376	FIGHT-202 [100]	Pemigatinib	2nd line or later, advanced CCA with or without *FGFR2* fusions, rearrangement, or alteration	ORR with *FGFR2* fusions, rearrangement	6.9	(6.2, 9.6)	21.1	(14.8, NE)	35.5%
NCT02052778	FEONIX-CCA2 [101]	Futibatinib	2nd line or later, advanced iCCA with *FGFR2* fusions, rearrangement	ORR	9.0	(6.9, 13.1)	21.7	(14.5, NE)	42%
NCT04083967	RAGNAR [102]	Erdafitinib	2nd line or later, advanced solid tumors with *FGFR1-4* alterations	ORR	5.2	(4, 5.6)	10.9	(7.9, 14.3)	CCA: 41.9%
NCT04526106	ReFocus [103]	RLY-4008	2nd line or later, advanced CCA with *FGFR2* fusion, rearrangement	ORR	Ongoing				88.2%
IDH1									
NCT02989857	ClarIDHy [104, 105]	Ivosidenib vs placebo	2nd line or later, advanced CCA with *IDH1* mutation	PFS	6.9 vs 2.7	0.37 (0.25, 0.54) *p* < 0.0001	10.3 vs 5.1	0.49 (0.34, 0.70) *p* = <0.001	2% vs 0%
NTRK									
NCT02097810 NCT02568267 EudraCT 2012-000148-88	STARTRK-1/2, ALKA-372-002 [106]	Entrectinib	2nd line or later, advanced solid tumors, *NTRK*-fusion positive	ORR, DOR	13.8	(10.1, 20.0)	37.1	(27.2, NE)	61.3%
NCT02122913 NCT02637687 NCT02576431	LOXO-TRK-14001, SCOUT, NAVIGATE [107]	Larotrectinib	2nd line or later, advanced solid tumors, *NTRK*-fusion positive	ORR	28.3	(22.1, NE)	44.4	(36.5, NE)	79%
RET									
NCT03157128	LIBRETTO-001 [108]	Selpercatinib	2nd line or later, advanced solid tumors, *RET*-fusion positive	ORR	13.2	(7.4, 26.2)	18.0	(10.7, NE)	43.9%
NCT03037385	ARROW [109]	Pralsetinib	2nd line or later, advanced solid tumors, *RET*-fusion positive	ORR	7.4	(5.1, 13.6)	13.6	(7.5, NE)	57.%

Abbreviations: *BTC*, biliary tract cancer; *CCA*, cholangiocarcinoma; *CRT*, chemoradiotherapy; *DOR*, duration of response; *eCCA*, extrahepatic cholangiocarcinoma; *GBC*, gallbladder cancer; *ORR*, objective response rate; *mOS*, median overall survival; *mPFS*, median progression-free survival; *NE*, not evaluable

### Second line — targeted systemic therapy

The development of targeted therapeutic agents with the potential for less systemic side effects and improved efficacy have expanded treatment options for patients with advanced eCCA. Figure 1 illustrates the most frequent actionable mutations found in BTC and Table 2 summarizes key clinical trials in target-selected populations. Herein, we will address the most frequently found mutations in eCCA, acknowledging that rare genetic alternations found in eCCA such as *IDH1/2 mutations* and *FGFR2*, *NTRK*, and *RET* fusions have targetable therapeutic options but are found almost exclusively in iCCA and will not be discussed in depth below but are included as part of Table 2 [6].

#### HER2/HER3

Human epidermal growth factor receptor 2 (HER2[ERBB2]) amplifications or mutations are found in 10–15% of eBTC with HER2 overexpression reported at even higher frequencies [23, 30, 110]. At this time, this represents the most frequently altered molecular target with effective therapeutic options that is unique to eBTC subtypes compared to iCCA.

In the phase II SUMMIT basket trial [111], eCCA patients harboring *HER2* mutations received neratinib, an oral pan-HER small molecule tyrosine kinase inhibitor (TKI), monotherapy daily (28-day cycles). Among cholangiocarcinoma patients, PFS (mPFS 1.4 months, [95% CI 0.5, 9.1]), OS (mOS 5.4 months, [95% CI 0.8, 16.2]), and ORR (16%) were modest in this pretreated population [87]. The most notable grade 3+ AE was diarrhea in 24% of patients [111].

Dual HER2 inhibition with trastuzumab and pertuzumab (anti-HER2 antibodies) has also been studied in advanced BTC with HER2 amplification/overexpression. In the phase IIa MyPathway [88] HER2-targeted BTC study (*N* = 39: *n* = 12 [eCCA/ampullary cancer]), BTC patients with HER2 amplification and/or overexpression (measured by next-generation sequencing, IHC ± FISH) who received pertuzumab + trastuzumab (21-day cycles) had mOS of 10.9 months (95% CI 5.2, 15.6) and mPFS of 4.0 months (95% CI 1.8, 5.7). ORR was 23%, and mDOR was 10.8 months. Patients tolerated therapy well with minimal AEs (grade 3/4 of AST elevation [13%], ALT elevation [13%], and hyperbilirubinemia [11%]) [88].

The antibody-drug conjugate trastuzumab-deruxtecan (T-DXd) showed improvement in ORR (36.4%; 90% CI 19.6, 56.1; *p* = 0.01) in patients with HER2-expressing BTC (*n* = 6 [eCCA]) in the phase II HERB trial [89]. Median PFS and mOS were 4.4 months (95% CI 2.8, 8.3) and 7.1 months (95% CI 4.7, 14.6), respectively. It is significant to note that 81.3% of patients had at least grade 3 AEs (most commonly anemia, neutropenia, leukopenia), and 25% developed interstitial lung disease. The DESTINY-Pantumor02 trial [90] is currently in progress, but interim results show a promising ORR of 22% in BTC with DOR of 8.6 months (95% CI 2.1, NE) with T-DXd in patients with prior HER-2 therapy exposure. No new grade 3/4 AEs were reported, and the most common were neutropenia (19.1%), anemia (8.6%), and fatigue (6%) [89]. The final PFS and OS results are still maturing.

The ongoing phase II HERIZON-BTC-01 trial [91••] is evaluating zanidatamab, a HER2 bispecific antibody targeting multiple HER2 domains, in 87 BTC patients (18% eCCA) with either IHC 2+/3+ (*N* = 80 patients) or IHC 0/1+ (*N* = 7 patients). The ORR in HER2+/3+ patients was 41.3% (79.4% and 11.8% in HER 3+ and 2+ patients, respectively) with a median DOR of 12.9 months. Median PFS was 5.5 months (95% CI 3.7, 7.2) with OS not yet reported. Grade 3/4 AEs were reported in 18% of patients, most commonly diarrhea (5%) and decreased ejection fraction (3%) [91••]. With no responses in patients with HER2 0/+1, this suggests no current role for HER2-directed therapy in HER2-low BTC.

The combination of the HER2-selective TKI, tucatinib, with trastuzumab was studied in HER2+ amplified/overexpressing BTC post 1L GemCis in the phase 2 SGNTUC-019 study [92]. The ORR of 46.7% is promising in this pre-treated population with reported DOR of 6 months (90% CI 5.5, NE). The PFS of 5.5 months (90% CI 3. 9, 8.1) is similar to the PFS benefit from zanidatamab. Grade 3/4 AEs of cholangitis, anorexia, and nausea were reported in 10% of patients [92].

Overall, there are several exciting options for HER2-overexpressing BTC. Given the proportion of eCCA patients with HER2 overexpression or amplification, early molecular testing is paramount as ongoing trials are needed to evaluate the benefit of targeted treatment in the front-line setting. Further standardization of HER2-positive criteria, along with prospective data on 1L use and in patients previously exposed to HER2 therapy to evaluate sequencing of these agents, will be important areas of inquiry going forward.

#### BRAF

Downstream of EGFR and HER2, the MAPK pathway consisting of the RAS-RAF-MEK-ERK signal cascade participates in a series of activation events ultimately leading to cellular proliferation and when disrupted, tumorigenesis [112]. BRAF mutations, more commonly found in iCCA (2–10%), are present in just 1–2% of eCCA and tend to be a poor prognostic indicator [19, 113]. The phase II ROAR basket trial [93] evaluated the efficacy of dual BRAF/MEK inhibition with dabrafenib (oral BRAF inhibitor) and trametinib (oral MEK inhibitor) in 43 (20.9% of study population) patients with BTC and BRAF^V600E^ mutations. All patients in the BTC cohort had been on prior therapy. The medication was very well tolerated with grade 3/4 AEs of elevated γ-glutamyl transferase and leukopenia in 3% of patients. The ORR in the BTC cohort was 47% with a DOR of 8.9 months (95% CI 5.6, 13.7). The mPFS of 9 months (95% CI 5.5, 9.4) and mOS of 13.5 months (95% CI 10.4, 17.6) are promising results in heavily pre-treated patients [93].

#### KRAS

Though RAS mutations have been reported in 37% of patients with eCCA, actionable RAS mutations such as KRAS^G12C^ are much rarer, occurring in 1–2% of BTC [25•, 114]. The phase II KRYSTAL-1 trial [94] evaluated adagrasib, an oral KRAS^G12C^ irreversible inhibitor, in 12 patients (21% of study population) with BTC. The BTC cohort had been on a median of 1.5 lines of prior therapy and showed promising results in ORR (41.7%; 95% CI 15.2, 72.3), mPFS (8.6 months; 95% CI 2.7, 11.3), and mOS (15.1 months; 95% CI 8.6, NE). The DOR for the entire BTC cohort was 5.3 months (95% CI 2.8, 7.3). Reported grade 3/4 AEs were fatigue (11.1%), anemia (7.9%), and QT prolongation on EKG (6.3%) [94]. A phase I/II clinical trial is underway evaluating the new KRAS^G12D^ inhibitor, MRTX1133, in solid tumors [115]. Recently, a novel pan-KRAS inhibitor (BI-2493) showed efficacy in animal models and its appearance in clinical trials is highly anticipated for KRAS-mutated tumors [116].

#### EGFR

EGFR dysregulation, reported in up to 15% of eCCA, allows growth and metastasis by aberrant signaling through the MAPK pathway [19, 117]. Though EGFR inhibition seems promising, studies have failed to produce significant differences in outcomes with EGFR-targeted therapies. The phase II BINGO [95] trial in 2014 compared GemOx ± cetuximab (anti-EGFR monoclonal antibody) in 150 patients with advanced BTC (*n* = 22 [eCCA]). ORR was similar between the 2 cohorts (18% [GemOx + cetuximab] vs 17% [GemOx]) with median DOR of 5.7 months (GemOx + cetuximab) vs 8.4 months (GemOx). There was no significant difference in OS (mOS 12.4 months [GemOx + cetuximab] vs 11 months [GemOx]). Interestingly, KRAS, BRAF mutation, or EGFR overexpression were not prognostic. Subsequently, patients were stratified by KRAS mutation status to GemOx ± cetuximab. While there was a numerical trend towards improved PFS in the GemOx + cetuximab KRAS-wild type arm, this was not significant (mPFS 7.1 months [GemOx + cetuximab] vs 5.6 months [GemOx]; *p* = 0.06) [118]. Similarly, a phase III study [96] evaluating GemOx ± erlotinib (oral EGFR TKI) failed to produce a significant difference in OS (mOS 9.5 months in both arms, HR 0.93 [95% CI 0.69, 1.25], *p* = 0.611) or PFS (mPFS 4.2 months [GemOx] vs 5.8 months [GemOx + erlotinib], HR 0.80 [85% CI 0.61, 1.03], *p* = 0.087). A subgroup analysis showed improvement in PFS for patients with CCA who received GemOx + erlotinib (5.9 months [GemOx + erlotinib] vs 3 months [GemOx]; HR 0.73 [95% CI 0.53, 1.0], *p* = 0.049). Unfortunately, lack of significant benefit has also been shown in studies evaluating panitumumab. Currently, there is no role for EGFR-inhibition in the treatment of eCCA [119, 120].

#### BRCA1/2

BRCA mutations leading to DNA damage repair deficiency have been reported in 2–5% of eCCA (similar BRCA1/2 incidence) [121]. Though studies evaluating the therapeutic implications of BRCA mutations are ongoing in BTC [122], their response to platinum chemotherapy and poly-ADP ribose polymerase inhibitors (PARPi) has been shown in several other cancer types, including pancreas cancer [123]. Since platinum agents are currently recommended in both the 1L and 2L setting for advanced BTC, further studies evaluating PARPi, immunotherapy, and their combination are needed for patients following platinum-based therapy. Genomic instability from BRCA mutations has been significantly associated with higher tumor mutational burden (TMB), regardless of deficient or proficient mismatch repair (dMMR or pMMR) [121]. Given that BRCA mutations are often associated with dMMR/MSI-H, the combination of PARPi + ICI could aid in the prevention of PARPi resistance and improve outcomes [124]. This combination is currently being studied in advanced BTC in the phase II trial combining rucaparib (PARPi) with nivolumab after proven platinum sensitivity (no radiographic or clinical progression after 4–6 months of platinum-based systemic chemotherapy), the results of which are anticipated in 2024 [125].

#### Microsatellite instable (MSI-H)/mismatch repair deficient (dMMR)

The prevalence of dMMR is estimated to be 5–13% in eCCA [126, 127]. The phase II KEYNOTE-158 [128] trial evaluated 22 (9.4% of total study population) patients with MSI-H CCA to receive pembrolizumab for up to 2 years or disease progression. The ORR of 40.9% (95% CI 20.7, 63.6%) in the CCA cohort with a DOR for all cancer types of 47.5 months (95% CI 2.1+, 51.1+, indicating no PD at the time of final analysis) in the updated analysis is especially promising in the 2L setting [97]. In the CCA group, the mPFS was 4.2 months (95% CI 2.1, NE) and mOS 24.3 months (95% CI 6.5, NE) [97]. Though grade 3/4 immune-related adverse events (IRAEs) of pneumonitis (1.3%), skin reaction (1.3%), colitis (0.9%), and hepatitis (0.9%) were reported, ICI in patients with dMMR/MSI-H CCA can improve survival by years for patients with an otherwise dismal prognosis on 2L therapy [128]. Because of this, pembrolizumab was given a tumor-agnostic FDA indication in 2017 for dMMR/MSI-H solid tumors.

#### High tumor mutation burden (TMB-H)

TMB has emerged with sequencing to quantify the number of mutations per megabase (mt/MB) of DNA to predict response to certain therapies and serve as a prognostic indicator. Because a higher mutational burden produces neoantigens that can be recognized by the immune system, those with very high TMB (>50 mt/MB) tend to have a better prognosis than patients with intermediate TMB given improved immune response and tumor infiltration [129]. Similarly, a linear correlation has been observed in a wide range of solid tumors between TMB and response to ICI such that as TMB increases, response to ICI increases [130]. High TMB (TMB-H; ≥10 mt/MB) has been reported in up to 8.2% of CCA [131]. In BTC, TMB-H (>20 mt/MB) often co-exists with *TP53* (58%) and DNA damage repair mutations such as BRCA1/2 and dMMR (78%) [132]. In TMB-H CCA patients who received ICI, ORR were significantly improved (25% vs 13.5%; *p* = 0.048) [131]. In the Checkmate-848 phase II trial [98], including over 40 TMB-H (≥10mt/MB) solid tumor types, dual ICI with ipilimumab + nivolumab (ipi + nivo) produced an ORR of 35.3% (95% CI 24.1, 47.8%), mPFS of 4.1 months (95% CI 2. 8, 11.3), and mOS of 14.5 months (95% CI 7.7, NE) [48]. Accordingly, ipi + nivo can be considered for TMB-H patients in the second or later line. Further quantifying TMB with molecular profiling up-front can help predict response to immunotherapy, especially since TOPAZ-1 and KEYNOTE-966 trials have added ICI to the 1L setting. Those with a low TMB (<10 mt/MB) may not derive benefit from the addition of ICI and may be subjected to increased risk of IRAEs. Continued molecular profiling of targetable co-existing mutations along with risk stratifying TMB to assess response to ICI is needed to further evaluate the treatment landscape in eCCA.

#### L-type amino acid transporter (LAT1)

More recently, LAT1 has been identified as a promising target for therapy in advanced BTC. LAT1, found at low levels in non-cancer cells, is over-expressed in various cancers due to increased pathologic metabolic requirements and has been shown to be associated with poor survival [133–135]. A phase II trial [99••] compared nanvuranlat (LAT1 inhibitor) to placebo in patients with advanced, chemo-experienced BTC (*N* = 104; *n* = 15 [eCCA]). Treatment with LAT1 inhibition significantly improved PFS compared to placebo (HR 0.59 [95% CI 0.34, 0.90], *p* = 0.016). Notably, this was driven by patients with LAT1-high tumors (HR 0.44 [95% CI 0.23, 0.85], *p* = 0.013) who made up over two-thirds of the total study population (*n* = 65 [LAT1-high], *n* = 32 [LAT1-low]). These LAT-high patients also trended towards improved OS with Nanvuranlat treatment. LAT1 inhibition did not improve outcomes patients with LAT1-low BTC. The greatest PFS benefit was seen in the eCCA (HR 0.15 [95% CI 0.04, 0.52]) and GBC (HR 0.26 [95% CI 0.10, 0.82]) cohorts, likely due to Nanvuranlat’s pharmacokinetics with its active metabolites attaining a high extrahepatic biliary concentration [99••, 136]. LAT1 expression status stratified by tumor site is not available so the question as to whether eCCA and GBC tumors were more likely to be LAT1-high expressors (compared to iCCA) is not known. Overall, Nanvuranlat was well tolerated with minimal treatment-related grade 3/4 AEs, likely owing in large part to its pharmacokinetics that show limited presence of drug systemically [136]. The future of this agent will likely be in biomarker-select populations and as part of a combinatorial regimen rather than monotherapy.

## Palliative considerations and supportive care

Patients with eCCA not only have symptoms associated with their cancer that may impact their quality of life (QOL) such as jaundice, pruritis, and chronic abdominal pain, but they may also experience acute and chronic treatment-related toxicities such as peripheral neuropathy with platinum-based treatment. With the addition of ICI in the 1L setting, patients may suffer from arthralgias or dermatitis, and even more severe IRAE such as pneumonitis, colitis, and hepatitis. Both chemotherapy and underlying illness can result in significant fatigue, nausea, and/or anorexia-cachexia which can all have significant impact physical/functional and psychosocial quality of life. Nutritional support from dieticians, patient and family support with psychotherapy or counseling, and early co-management with palliative care specialists to address unmet needs and conduct iterative serious-illness conversations are fundamental to providing comprehensive care for patients with eBTC.

In patients with malignant biliary obstruction, up to 26.5% can develop cholangitis prior to stenting with a 30-day mortality rate up to 30.8% [137]. Palliative endobiliary stenting during ERCP or by placing a percutaneous biliary drain may alleviate symptoms while preventing cholangitis, but also requires intermittent stent exchange [138]. The decision to pursue ERCP or percutaneous drainage should be made with the input of both interventional radiology and surgical oncologists. Alongside stenting, both photodynamic therapy (PDT) and endobiliary radiofrequency ablation (RFA) are used to alleviate obstruction or as a bridge to surgery. PDT and RFA may be performed either endoscopically or percutaneously with interventional radiology [139]. Biliary stenting with either eRFA or PDT has been associated with longer stent patency than endobiliary stenting alone with improved patient-reported QOL and may also have a dual effect of improving response to chemotherapy [140–142].

## Conclusion

Extrahepatic BTCs are diverse cancers with distinct molecular features but share an overall poor prognosis. With the addition of ICI and growing identification of actionable mutations paired with targeted therapy development, patients with eCCA may have more treatment options beyond standard chemotherapy. However, patients with eCCA are less likely to receive upfront molecular profiling (50.5% [eCCA] vs 64.3% [iCCA]; *p* < 0.001) than patients with iCCA, which may be in part due to difficulty obtaining enough tissue on diagnosis (especially if this is done with ERCP) [25•]. Fortunately, with the development of blood-based circulating tumor DNA testing, targetable mutations can be evaluated upfront even without tumor tissue. Given the high mortality rate for patients with advanced eCCA, patients with refractory disease may not be appropriate candidates for continued therapy with targeted agents even if they had identified mutations. Reportedly, only 15–40% of patients have a performance status after 1L therapy appropriate for 2L therapy [143]. Therefore, more prospective data is needed to see if there is benefit to incorporating targeted agents earlier in the treatment course or in combination with chemotherapy or ICI for additive/synergistic/complimentary effect. Continued upfront molecular testing, early clinical trial enrollment, and integration of multidisciplinary management are critical factors in improving survival and optimize quality of life for patients with eCCA.
